# The Cape Town Declaration on Access to Cardiac Surgery in the Developing World

**DOI:** 10.5830/CVJA-2018-046

**Published:** 2018

**Authors:** Zilla Peter, Bolman R Morton, Yacoub Magdi H, Beyersdorf Friedhelm, Sliwa Karen, Zühlke Liesl, Higgins Robert SD, Mayosi Bongani, Carpentier Alain, Williams David

**Affiliations:** Christiaan Barnard Division of Cardiothoracic Surgery, Faculty of Health Sciences, University of Cape Town, Cape Town, South Africa; Division of Cardiothoracic Surgery, University of Vermont, Burlington, Vermont, USA; Chain of Hope, Chelsea, London, United Kingdom; Department of Cardiovascular Surgery, Universitats- Herzzentrum Freiburg–Bad Krotzingen, Freiburg, Germany; Hatter Institute of Cardiovascular Research in Africa, Faculty of Health Sciences, University of Cape Town, Cape Town, South Africa; Division of Paediatric Cardiology, Department of Paediatrics and Child Health, University of Cape Town, Cape Town, South Africa; Department of Surgery, Johns Hopkins Medicine, Baltimore, Maryland, USA; Faculty of Health Sciences, University of Cape Town, Cape Town, South Africa; Hôpital Européen Georges Pompidou, Paris, France; Wake Forest Institute of Regenerative Medicine, Wake Forest School of Medicine, Winston-Salem, North Carolina, USA

## Abstract

**Mission**: to urge all relevant entities within the international cardiac surgery, industry and government sectors to commit to develop and implement an effective strategy to address the scourge of rheumatic heart disease in the developing world through increased access to life-saving cardiac surgery.

Twelve years after cardiologists and cardiac surgeons from all over the world issued the Drakensberg Declaration on the Control of Rheumatic Fever and Rheumatic Heart Disease in Africa, calling on the world community to address the prevention and treatment of rheumatic heart disease (RHD) through improving living conditions, to develop pilot programmes at selected sites for control of rheumatic fever and rheumatic heart disease, and to periodically review progress made and challenges that remain,^1^ RHD still accounts for a major proportion of cardiovascular diseases in children and young adults in low- and middle-income countries, where more than 80% of the world’s population live. Globally equal in prevalence to human immunodeficiency virus infection, RHD affects 33 million people worldwide.^2^

Prevention efforts have been important but have failed to eradicate the disease. At the present time, the only effective treatment for symptomatic RHD is open-heart surgery, yet that life-saving cardiac surgery is woefully absent in many endemic regions. In this declaration, we propose a framework structure to create a co-ordinated and transparent international alliance to address this inequality.

Elimination of RHD and relief from its debilitating consequences can only occur through interdisciplinary effort, as outlined in the Cairo Accord.^3^ Previous initiatives have focused on primary and secondary prevention of RHD.^4^ Their declarations have been recognised by the heads of state of African Union countries and by the World Health Organisation. This recognition has been important in developing recommendations by the World Health Organisation executive board to the 2018 World Health Assembly to enlist global commitment to RHD.

Progress in the prevention of RHD has been slow during the past 15 years,^5^ and therefore surgery will likely remain an integral part of RHD treatment for several generations. Lack of access to cardiac surgery services and the cost of valve replacement render this disease fatal for millions of patients. In endemic regions of low-income countries, the need for cardiac surgery is estimated at 300 operations per one million population (Global Unmet Needs in Cardiac Surgery, unpublished work by Zilla and colleagues), yet, the nearly one billion people living in sub-Saharan Africa between the Maghreb and South Africa have access to only 22 cardiac centres.^6^

Although there is one cardiac centre per 120 000 people in the United States, there is only one centre per 33 million in Africa. Furthermore, RHD is not restricted to sub-Saharan Africa. India, Pakistan, China and Indonesia together account for 72% of the mortality rate of RHD cases worldwide.^2^

We strongly endorse the position that building local capacity is the best solution for this serious public health problem. Many lives have been saved by humanitarian ‘fly-in’ missions, but these efforts are neither sustainable nor cost effective. The non-governmental organisations associated with these programmes are shifting focus towards building long-term partnerships with host countries to develop autonomous local services with government buy-in.^7^ A massive investment in new cardiac centres in these regions is unrealistic; globally, an additional few thousand cardiac centres would be required to address the unmet needs (Global Unmet Needs in Cardiac Surgery, unpublished work by Zilla and colleagues).

It is not sufficient for governments and non-governmental organisations to support the training of cardiologists and cardiac surgeons from these regions at high-income country facilities, because they will not be trained in most of the pathologies awaiting them in their own countries and will be unfamiliar with resource-constrained circumstances.

There is an urgent need for a concerted effort by all stakeholders to address the plight of the poor in these regions, who need cardiac surgery. As signatories and endorsing organisations of the Cape Town Declaration, we propose a comprehensive solution with two principal aims.

**Aim 1: To establish an international working group (coalition) of individuals from cardiac surgery societies and representatives from industry, cardiology and government to evaluate and endorse the development of cardiac care in low- to middle-income countries.**

It is proposed that the international coalition will have two representatives from each of the major cardiac surgery societies (the Society of Thoracic Surgeons, American Association for Thoracic Surgery, European Association for Cardio-Thoracic Surgery, the Asian Society for Cardiovascular and Thoracic Surgery), and ideally, two additional committed members. There will be at least one representative from industry and at least one appointee to represent cardiology/the World Heart Federation. The responsibilities of the coalition will include establishing criteria for centres for clinical care and training as well as selecting and endorsing the centres. The coalition will derive metrics of quality and performance for the endorsed centres of training and clinical care and will encourage standardisation of care to the extent possible.

The coalition will advocate mutually agreed policies and prescriptions to relevant governmental bodies. In addition, the coalition will engage with industry and private sources of philanthropy for financial assistance with large-scale initiatives.

**Aim 2: To advocate for the training of cardiac surgeons and other key specialised caregivers at identified and endorsed centres in lowto middle-income countries.**

The case has been made above for critical providers obtaining training in settings and conditions and dealing with the cardiac pathologies that they will be encountering in their practice in their countries of origin.

It is preferred that centres endorsed by this coalition be based on an alliance of four stakeholders: a programme initiator (e.g. a government, a university, or a non-governmental organisation), an audited training centre in a low- to middle-income country, a committed partner institution in a high-income country, and a consortium of industry that would sign on as benefactors to the specific programme. Because regional centres in low- to middleincome countries typically operate within a resource-scarce environment, resulting in lower case numbers than needed for the training of outside residents, a facilitated capacity increase to help achieve higher case numbers would benefit all participants.

**Summary: It is imperative that action be taken urgently. A nucleus of one to three centres should be identified and endorsed, with co-ordination by global stakeholders, as quickly as possible. The implementation of this initiative will only be made possible by the endorsement of all the relevant cardiothoracic societies and agencies subscribing to clearly defined targets and timelines, and committing appropriate resources. The time to act is now.**

## Signatories

For Cardiothoracic Societies (in alphabetical order)

Joseph Bavaria [past-president, the Society of Thoracic Surgeons (STS), USA]
Friedhelm Beyersdorf (editor in chief, European Journal of Cardio-Thoracic Surgery, Germany)
R Morton Bolman, III [representative, American Association for Thoracic Surgery (AATS), USA]
Kumud Dhital [representative, Australian and New Zealand Society of Cardiac and Thoracic Surgeons (ANZSCTS), Australia]
Robert SD Higgins [president elect, the Society of Thoracic Surgeons (STS), USA]
James Kirklin [representative, American Association for Thoracic Surgery (AATS), USA]
Robert Kleinloog [president, Society of Cardiothoracic Surgeons of South Africa (SCTSSA), South Africa]
Bongani Mayosi [past president, Pan-African Society of Cardiology (PASCAR), South Africa]
Juan Mejia (representative, Brazilian Society of Cardiovascular Surgery, Brazil)
Jose Pomar [past president, European Association for Cardio- Thoracic Surgery (EACTS), Spain]
Karen Sliwa [president elect, World Heart Federation (WHF), South Africa]
Shinichi Takamoto [president, Asian Society for Cardiovascular and Thoracic Surgery (ASCVTS), Japan]
Wei Wang (representative, Chinese Society for Thoracic and Cardiovascular Surgery, China)
David Wood [president, World Heart Federation (WHF), United Kingdom]
Charles Yankah (president, Pan-African Society for Cardiothoracic Surgery (PASCaTS), Ghana/Germany]
Liesl Zuhlke (president, South African Heart Association, South Africa)

Humanitarian and government organisations (in alphabetical order)

Alain Carpentier (Alain Carpentier Foundation, France)
Sylvain Chauvaud (La Chaîne de l’Espoir, France)
Afksendiyos Kalangos (President: Kalangos Foundation, Greece)
Richard Kamwi (Past Minister of Health, Namibia)
René Prêtre (Le Petit Coeur, Switzerland)
Nicole Sekarski (Le Petit Coeur, Switzerland)
Magdi Yacoub (President, Chain of Hope, United Kingdom/Egypt)

Industry and health economy representatives (in alphabetical order)

Iraj Abedian (Pan-African Capital Holdings, South Africa)
Francois Bonnici (director, Bertha Centre for Social Innovation and Entrepreneurship, South Africa)
Lenias Hwenda (chief executive officer, Medicines for Africa, Zimbabwe/Switzerland)
Sidhant Jena (chief executive officer, Jana Care, USA)
Samukeliso Dube (chief medical officer, Royal Philips, Netherlands)
Jacques Kpodonu (author, Global Health Innovations, USA/ Ghana)
Salah Malek (president, Middle East and Africa, Getinge, United Arab Emirates)
Markus Stirner-Schilling (VP Marketing and Academy, Europe, Middle East and Africa, Getinge, Germany)
Patrice Matchaba (global head corporate responsibility, Novartis, Switzerland)
Lee Chuen Neng (Biomedical Institute for Global Health Research and Technology, Singapore)
Michael P Phalen (executive president, MedSurg Boston Scientific, USA)
Tim Ring (chairman and chief executive officer, CR Bard Inc, USA)
Kathryn Gleason (co-founder, TeamFund Inc)
Devi Prassad Shetty (NH Group, India)
Alistair Simpson (general manager cardiac surgery, LivaNova, United Kingdom)
Raenette Taljaard [Economic Research of South Africa (ERSA), South Africa]
Helmut Straubinger (ex-chief executive officer, Jena Valve; chief executive officer, Tricares, Germany)
Barry Wilson (former president, Medtronic International)

For major journals and publications (in alphabetical order)

Friedhelm Beyersdorf (editor in chief, European Journal of Cardio-Thoracic Surgery, Germany)
Sampath Kumar (editor in chief, Asian Cardiovascular and Thoracic Annals, India)
Dan Ncayiyana (past editor in chief, South African Medical Journal, South Africa)
JP van Niekerk (past editor in chief, South African Medical Journal, South Africa)
G Alexander Patterson (editor in chief, The Annals of Thoracic Surgery, USA)
Marko Turina [past editor in chief, European Journal of Cardio- Thoracic Surgery; past editor in chief, Cardiothoracic Surgery Network (CTSNet) and Multimedia Manual of Cardio- Thoracic Surgery, Switzerland]
David Williams (past editor in chief, Biomaterials, USA)

Other signatory delegates to the Cape Town Dialogue (in alphabetical order)

Elena Aikawa (USA)
Masanori Aikawa (USA)
Henning Rud Andersen (Denmark)
Manuel Antunes (Portugal)
Eduardo Becerra (Chile)
Solomon Benatar (South Africa/Canada)
Richard Bianco (USA)
Bojan Biocina (Croatia)
Carlijn Bouten (Netherlands)
Abdelmalek Bouzid (Algeria)
Luke Brewster (USA)
Johan Brink (South Africa)
Taweesak Chotivatanapong (Thailand)
Patrick Commerford (South Africa)
David Cooper (USA)
John Curci (USA)
Manfred Deutsch (Austria)
Richard Daly (USA)
Frederick C de Beer (USA)
Norberto de Vega (Spain)
Howard Eisen (USA)
Max Emmert (Switzerland)
Giuseppe Faggian (Italy)
Volkmar Falk (Germany)
Ted Feldman (USA)
Giovanni Ferrari (USA)
Teddy Fischlein (Germany)
Robert Frater (USA)
Glen Gaudette (USA)
Gino Gerosa (Italy)
Bernard Gersh (USA)
Allan Glanville (Australia)
Craig Goergen (USA)
Claudia Goettsch (Germany)
Mervyn Gotsman (Israel)
Martin Grabenwoeger (Austria)
Andrea Griesmacher (Austria)
Christian Hagl (Germany)
Ulf Hedin (Sweden)
Simon Hoerstrup (Switzerland)
Saeid Hosseini (Iran)
Joshua Hutcheson (USA)
Willie Koen (South Africa)
Gennadiy Khubulava (Russia)
Robin Kleinloog (South Africa)
Walter Klepetko (Austria)
Michael Knaut (Germany)
Theodoros Kofidis (Singapore)
Guenther Laufer (Austria)
Nicolas l’Heureux (France)
Georg Lutter (Germany)
Simon Maltais (USA)
Simon Matskeplishvili (Russia)
Jo Meinhart (Austria)
Ana Olga Mocumbi (Mozambique)
Elmi Muller (South Africa)
Mathias Mueller (Austria)
Paul Mohacsi (Switzerland)
Ivan Netuka (Czech Republic)
Mpiko Ntsekhe (South Africa)
Minoru Ono (Japan)
Otmar Pachinger (Austria)
Timothy Pennel (South Africa)
Olga Plattner (Austria)
Bruno Podesser (Austria)
Darshan Reddy (South Africa)
Bruno Reichart (Germany)
Hermann Reichenspurner (Germany)
Hector Sanchez (Argentina)
Jacques Scherman (South Africa)
Stephan Schueller (United Kingdom)
Jim Schurman (USA)
Rainald Seitelberger (Austria)
Toshi Shinoka (USA)
Agneta Simionescu (USA)
Dan Simionescu (USA)
Francis Smit (South Africa)
Ulrich Steinseifer (Germany)
Cynthia St Hilaire (USA)
Justiaan Swanevelder (South Africa)
Hendrik Treede (Germany)
Nir Uriel (USA)
Devagourou Velayodamu (India)
David Vorp (USA)
Susan Vosloo (South Africa)
Beath Walpoth (Switzerland)
Georg Wieselthaler (USA)
Mike Wolf (USA)
Andreas Zuckermann (Austria)
